# INTERGENERATIONAL CO-PRODUCTION IN DIGITAL DESIGN FOR HEALTH, WELL-BEING, AND SOCIAL CONNECTION

**DOI:** 10.1093/geroni/igad104.2324

**Published:** 2023-12-21

**Authors:** Catherine Hennessy, Greg Mannion, Richard Haynes, Anna Whittaker, Hannah Bradwell, Simone Tomaz, John Ritchie, Leonie Cooper

**Affiliations:** University of Stirling, Stirling, Scotland, United Kingdom; University of Stirling, Stirling, Scotland, United Kingdom; University of Stirling, Stirling, Scotland, United Kingdom; University of Stirling, Stirling, Scotland, United Kingdom; University of Plymouth, Plymouth, England, United Kingdom; University of Stirling, Stirling, Scotland, United Kingdom; Communications, Media and Culture, Stirling, Scotland, United Kingdom; University of Plymouth, Plymouth, England, United Kingdom

## Abstract

The benefits of intergenerational practice in bringing different generations together in initiatives and programs to address issues like social isolation and loneliness are well-established. Less frequently employed as a vehicle for using intergenerational contact in the development of interventions and design solutions, intergenerational co-production involving older and younger people with academics and designers, has rarely been tried. Thus, intergenerational co-production is not common and is under-theorised despite holding significant promise for advancing societies for all ages. This paper reports on the experience of intergenerational co-production in the GOALD (Generating Older Active Lives Digitally) project, an interdisciplinary collaboration in Scotland and England using intergenerational co-production methods in the creation of digital tools to support older adults’ health, well-being and social connectivity. It presents GOALD’s multi-year experience of constituting and running intergenerational co-production groups with geographically, socio-demographically and functionally diverse participants to provide feedback and design ideas to business partners for digital devices and applications for health promotion in later life. Mixed methods findings on sources, timing, locations and numbers of participants recruited and retained, their rates of participation, forms, frequency and modes of engagement, as well as organizational partners’ and participants’ motivations, incentives, capacity and constraints to take part demonstrate the barriers and facilitators of implementing intergenerational co-production, particularly during the Covid-19 pandemic. Our findings highlight lessons learned about the need for creative adaptation of this approach on the ground, and suggest GOALD’s potential contributions to new theorisation through enacting and understanding the features of intergenerational co-production and documenting its effects.

